# Mucinous Cystic Neoplasm of the Liver in a Teenager: A Case Report

**DOI:** 10.7759/cureus.65728

**Published:** 2024-07-30

**Authors:** Toshiyuki Moriuchi, Masakazu Hashimoto, Shintaro Kuroda, Tsuyoshi Kobayashi, Hideki Ohdan

**Affiliations:** 1 Department of Gastroenterological and Transplant Surgery, Graduate School of Biomedical and Health Science, Hiroshima University, Hiroshima, JPN

**Keywords:** hepatectomy, surgery, teenager, ovarian-like stroma, liver, mucinous cystic neoplasm

## Abstract

Mucinous cystic neoplasms of the liver (MCN-Ls) are rare cystic liver tumors. Herein, we report a case of MCN-L wherein complete surgical resection was successful. A 17-year-old girl initially presented to the referring hospital with a chief complaint of upper abdominal pain. Abdominal computed tomography (CT) revealed a cystic lesion in the medial segment of the liver. After eight months, the cystic lesion showed a tendency to increase in size, and the patient was referred to our hospital. CT showed a cystic lesion with dilation of the left hepatic duct and duct of the right anterior segment. Magnetic resonance imaging and abdominal ultrasonography revealed a multilocular cyst. Endoscopic retrograde cholangiopancreatography revealed sclerotic changes, dilatation, and irregular wall features in the left hepatic duct. No communication between the cystic lesion and the biliary system was observed; there was no evidence of biliary prolapse. A left hepatectomy and cholecystectomy were performed. Histological examination revealed an ovarian-like stroma (OLS); the lesion was diagnosed as MCN-L. The patient was recurrence-free six months postoperatively.To our best knowledge, this is the second reported case of teenage-onset MCN-L. We report the development of MCN-L in a teenager, highlighting the potential of this rare tumor for manifesting even at a young age. Our case demonstrated that MCN-L, despite its typically benign nature, should be carefully monitored. Although most cases of MCN-L do not require immediate surgery, timely surgical intervention may be necessary in cases of rapid growth or persistent symptoms.

## Introduction

Mucinous cystic neoplasms of the liver (MCNs) are classified into biliary cystadenomas or biliary cystadenocarcinomas. In 2010, the World Health Organization (WHO) reclassified mucin-producing bile duct tumors of the liver into two distinct types: MCN-Ls and intraductal papillary mucinous neoplasms (IPMN-Bs) [1]. Diagnosis of MCN-L requires the presence of ovarian-like stroma (OLS), which distinguishes MCN-L from IPMN-B. MCN-Ls are rare cystic tumors that arise within the liver parenchyma or, less frequently, within the extrahepatic bile ducts; they are reported to account for less than 5% of all hepatic cysts [2]. Although MCN-L is generally considered a benign tumor; malignant transformation can occur in rare cases [3]. Therefore, surgical intervention is recommended in patients presenting with a cyst size of 100 mm or greater, demonstrating a growth trend, and experiencing symptoms [4]. Complete surgical resection is associated with excellent long-term outcomes, achieving a five-year survival rate of 100%. The most important factor in preventing recurrence is achieving complete surgical resection without residual tumor [5,6]. These tumors predominantly occur in older women, typically aged 40-70 years [7]. Despite being extremely rare, instances of the disease have been documented in teenagers, indicating its potential onset at a young age [8]. To the best of our knowledge, there have been no detailed reports of cases necessitating surgical intervention in this age group; therefore, we present this case.

## Case presentation

**Figure 1:**
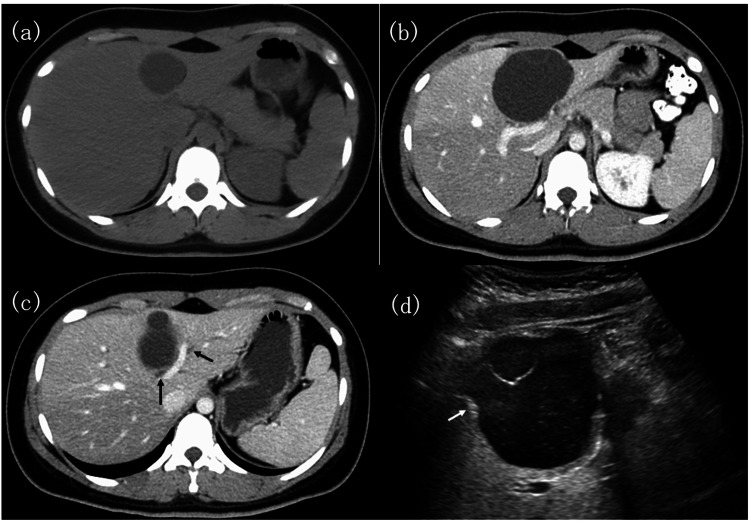
Abdominal computed tomography and abdominal ultrasonography findings a: Computed tomography (CT) showing a cystic lesion in the medial section of the liver measuring 35 mm. b: Contrast-enhanced CT showing a multi-ocular cystic tumor in the medial section of the liver with calcification in the cyst wall. The tumor measured 75 mm in diameter after 8 months. c: Dilation of the left hepatic duct and anterior segment bile duct (black arrow). d: Ultrasonography showing a multilocular cyst and a hyperechoic nodule of 6 mm in size (white arrow).

A 17-year-old female patient initially presented to the referring hospital with a chief complaint of epigastric pain. The patient had no relevant medical or family history. Abdominal computed tomography (CT) revealed a 35-mm cystic mass in the liver (Figure 1a). After eight months, the patient visited the same hospital with similar symptoms. Abdominal contrast-enhanced CT (CECT) revealed a cystic lesion with an increased diameter of 75 mm, exhibiting multilocular cystic formations in the medial segment of the liver (Figure 1b). The cyst had a thin septal structure. Since the cystic lesion was proximal to the root of the anterior section of Glisson, the left hepatic duct and anterior section of the bile duct were mildly dilated (Figure 1c). Abdominal ultrasonography revealed a multilocular cyst with a 6-mm hyperechoic nodule (Figure 1d). Magnetic resonance imaging revealed that the cystic lesion was hypointense on T1-weighted images and hyperintense on T2-weighted images.

**Figure 2:**
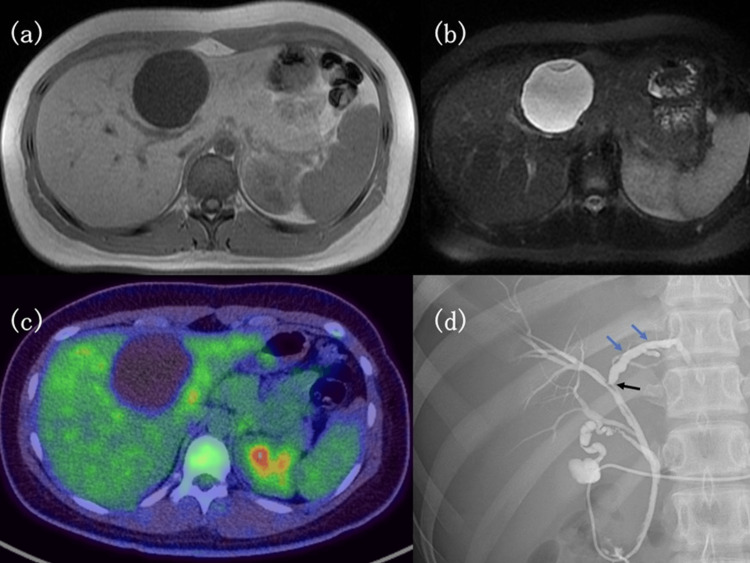
Imaging studies The figure shows Magnetic resonance imaging, Fluorodeoxyglucose-positron emission tomography/computed tomography, and Endoscopic retrograde cholangiopancreatography findings. a: The cystic lesion with a septum is hypointense on T1-weighted images. b: The cystic lesion is hyperintense on the T2-weighted images. c: The cystic lesion shows no fluorodeoxyglucose uptake. d: Mild compression of left hepatic biliary and anterior segment bilayers (black arrows). The left bile duct shows sclerotic changes and dilatation (blue arrows).

No solid components were found inside the cysts (Figures 2a, 2b). Fluorodeoxyglucose positron emission tomography-CT revealed no fluorodeoxyglucose uptake in the cystic lesion (Figure 2c). The lesion was symptomatic with a tendency to enlarge. Considering the inability to definitively exclude the malignant potential of the lesion, the patient was referred to our hospital for surgery. Endoscopic retrograde cholangiopancreatography (ERCP) demonstrated mild compression of the left hepatic duct and the duct of the right anterior segment. The left hepatic duct showed evidence of sclerosis, dilatation, and irregular wall features (Figure 2d). The duct of the right posterior segment originated at a lower level than usual. An endoscopic nasobiliary drainage (ENBD) tube was placed, and intraoperative cholangiography was planned to definitively identify and resect the left hepatic duct during surgery. No communication between the cystic lesion and the biliary system was observed, and there was no biliary prolapse. The patient’s functional liver reserve was good with a Child-Pugh score of 5 (A). Serum levels of alfa-fetoprotein (AFP), carbohydrate antigen 19-9 (CA19-9) and carcinoembryonic antigen (CEA) were within normal ranges. A left hepatectomy and cholecystectomy were performed, and the tumor was completely resected. The use of an ENBD tube for intraoperative cholangiography was highly beneficial, allowing for precise identification and resection of the left hepatic duct.

**Figure 3:**
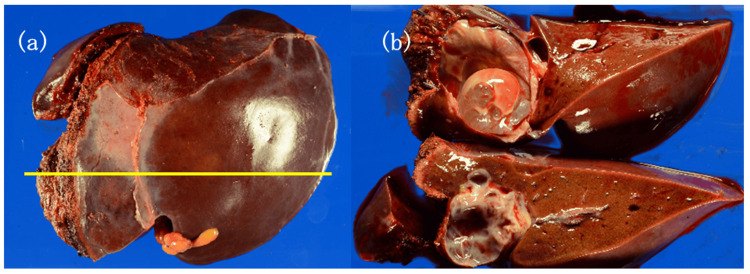
Macroscopic findings The cross-section revealed a multilocular cystic lesion with a smooth external surface, a thin wall with a smooth internal lining, and no solid components.

Macroscopic findings showed that the excised specimen had a multilocular cystic lesion with a smooth external surface and a thin wall with a smooth internal lining (Figures 3a, 3b). Although serum CA19-9 and CEA levels were within normal limits, cystic fluid levels of CA19-9 (12,000,000 U/mL) and CEA (61.1 ng/mL) were elevated. Cystic fluid levels of total bilirubin and amylase were within normal ranges. Cytological examination of the cystic fluid revealed negative results for malignancy. Cyst fluid collected from the excised specimen was sent to the laboratory.

**Figure 4:**
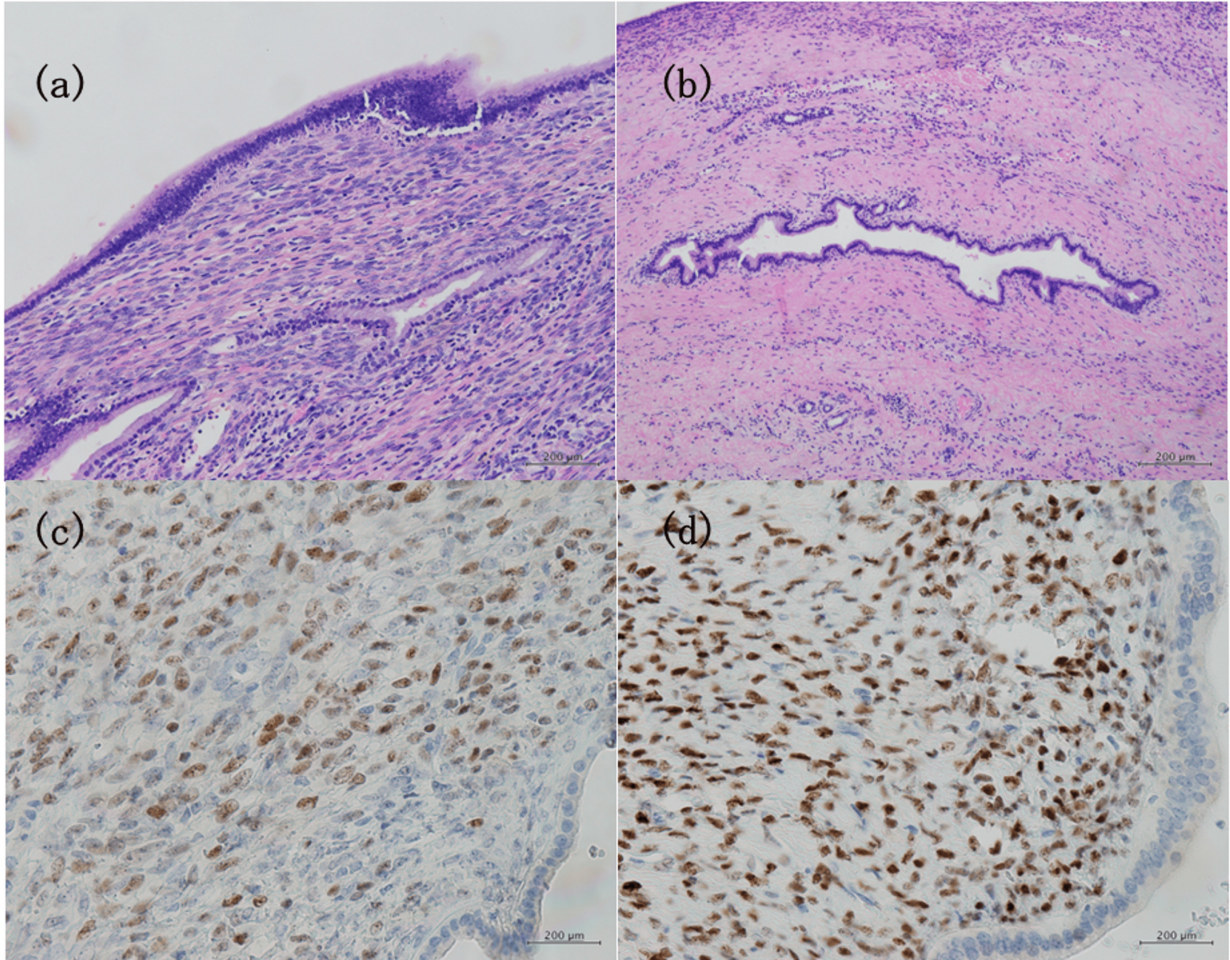
Histopathological findings and presentations of immunohistochemical stains a: The cyst was covered with a single layer of epithelial cells. Ovarian-like stroma was observed in the epithelium. b: Dilated left bile duct surrounded by mild inflammation. (hematoxylin-eosin staining, original magnification ×200); c: Estrogen receptor positivity (original magnification ×400). d: Progesterone receptor positivity (original magnification, ×400).

Microscopic findings revealed that the cyst comprised of mucus-secreting columnar and cuboidal epithelial cells. No evidence of malignancy was detected. The subepithelial tissue contained a layer of hypercellular OLS (Figure 4a). Lymphocytes and fibrosis were observed around the dilated left bile duct with mild inflammatory changes (Figure 4b). Immunohistochemistry (IHC) revealed that the OLS cells were positive for estrogen and progesterone receptors (Figures 4c, 4d). These findings supported the diagnosis of MCN-L. The patient was discharged 12 days after surgery without postoperative complications. Postoperatively, the patient was recurrence-free at the six-month follow-up. The patient is now asymptomatic and reports a good quality of life.

## Discussion

The WHO classifies mucin-producing bile duct tumors of the liver as MCN-L and IPMN-B. According to the 2010 WHO classification, MCN-L is considered a counterpart of mucinous cystic neoplasm of the pancreas (MCN-P) [1]. Shiono et al. reported that MCNs also occur in other organs, including the spleen and mesentery [9]. As in MCN-P, the etiology of MCN-L remains unclear. MCN-Ls mostly develop in the left lobe of the liver and predominantly occur in women aged 40-70 years [4,5]. Teenage-onset MCN-L is an extremely uncommon occurrence. To our best knowledge, this is the second reported case of teenage-onset MCN-L.

Differential diagnoses of MCN-L include IPMN-B, echinococcal cysts, simple liver cysts, and hemorrhage into a simple cyst [3,10,11]. Simple cysts are typically devoid of septations. However, distinguishing simple cysts from MCN-L can be challenging when a hemorrhage occurs in the cyst because imaging may reveal non-homogeneous cyst contents, septations, or a multilocular cyst appearance [12]. Imaging findings of MCN-L may be difficult to distinguish from those of echinococcosis, which shows calcified lesions and daughter nodules [3]. MCN-L is a cystic epithelial neoplasm of the liver that typically presents as a multifocal cystic tumor with a septate wall that does not communicate with the bile ducts. In contrast, IPMN-B involves communication with the bile ducts, dilation of these ducts; the presence of papillary projections in these ducts [11]. However, an accurate preoperative diagnosis of MCN-L and IPMN-B is difficult. Ultimately, excised specimens are essential for differentiating MCN-L from IPMN-B. Pathologically, the inner lining of the cyst comprises a single layer of mucus-secreting columnar, cuboidal, and squamous cells [13]. A final diagnosis is confirmed by the presence of an OLS, which is positive for estrogen, progesterone and inhibin-α on immunohistochemistry [14].

Most MCN-Ls manifest a cyst-in-cyst structure without bile duct communication. However, previous reports have shown communication between the cyst and bile ducts on intraoperative contrast [10] or bile duct dilatation on CT [15]. Furthermore, MCN-L manifesting bile duct dilatation and biliary prolapse has been reported [8]. MCN-L with biliary prolapse typically occurs in younger patients with smaller tumors and often manifests with obstructive jaundice. Characteristic findings on ERCP include an oval-shaped filling defect within the bile duct [8]. This case revealed bile duct dilation and sclerotic changes in the left hepatic duct, probably due to compression from the cyst. No evidence of biliary prolapse was observed. Microscopically, lymphocytes and fibrosis surrounding the dilated bile ducts suggested inflammation. Nakayama et al. [10], who reported a case of MCN-L with bile duct communication, along with studies on MCN-P [16], have proposed that the expanding cyst wall might erode into the bile duct, creating a fistula. Although bile duct dilation is a recognized finding in MCN-L, sclerotic changes within these dilated segments have not been reported. Further research and case accumulation are necessary to better understand this potential complication. In this case, cystic fluid levels of both CEA and CA19-9 were elevated. Regarding MCN-P, cystic fluid CEA levels are considered helpful in differentiating between mucinous cystic tumors [17]. However, a consensus regarding CEA and CA19-9 levels in cystic fluid for MCN-L remains elusive.

MCN-Ls are known to have a malignant potential, with documented cases of malignant transformation of the cystic epithelium [3]. Although less common than in MCN-P, MCN-L can be complicated by invasive cancer [18]. Therefore, surgical intervention is necessary for hepatic cystic tumors in some cases. Zen et al. noted that large cysts (>100 mm) at initial presentation, progressive enlargement during follow-up, and symptomatic manifestations are indications for surgical resection [4]. The preferred treatment of MCN-L is complete surgical resection to prevent recurrence or malignant transformation [2,3,16]. Although there have been no reports on drug therapies for preventing recurrence, achieving complete resection remains crucial. Lee et al. reported that most patients who underwent liver-preserving surgery, such as fenestration of the cyst wall (62.5%), experienced tumor recurrence [15]. Previous reports have suggested that the prognosis in patients with MCN-L who achieved complete resection was good, with a five-year survival rate of 100% [5,6]. This finding reinforces the importance of complete resection for optimal outcomes in MCN-L cases.

## Conclusions

We present a case of MCN-L in a teenage patient, highlighting the potential of this rare tumor for developing even at a young age. This case involved dilation and sclerosis of the left hepatic duct, probably due to cyst compression, necessitating surgical intervention due to the potential for malignant transformation. The patient, wherein a successful complete resection was achieved, has experienced a favorable outcome without evidence of recurrence. This case underscores the importance of regular follow-up, even in younger patients, as timely intervention could significantly improve prognosis.
